# Treating Roots

**Published:** 1889-04

**Authors:** Louis Ottofy

**Affiliations:** Chicago.


ARTICLE III.

### TREATING ROOTS.

DR. LOUIS OTTOFY, CHICAGO.

As a rule, the drilling, reaming, or any other method of enlarging root canals, is pernicious practice, and should be almost entirely abandoned. When a proper entrance to the pulp-chamber has been secured, before any effort has been made to enter the root-canals, the reaming or enlarging of the canal will not be found necessary. Opening into a pulp-chamber and securing an entrance into a root canal should always be done under perfect antiseptic precautions; for this purpose flooding the cavity with a one to two hundred solution of bichloride of mercury is efficacious.

Recently I have followed some interesting experiments conducted by Dr. G. V. Black regarding the antiseptic property of essential oils, and observing the property to be possessed in an extraordinary degree by the oil of cassia, which is very diffusible, I have been led to employ it for this purpose with satisfactory results. In filling the roots of the six anterior upper teeth, difficulties are seldom encountered, except from the occasional small size of the canal in the lateral incisor, which can readily be penetrated by a fine broach, if no foreign substance has been forced into it.

The rule that overhanging ledges should be cut away is inflexible when applied to all the teeth of the mouth, and it may be especially emphasized when the first upper bicuspids are in question; in the treatment of these teeth easy access is invariably necessary. The use of two broaches is good practice: introduce into the canal, which has been found, a large broach (as large a one as can be inserted), and, while leaving it there, endeavor to find another canal by means of a fine, stiff, oiled broach.

The upper first and second molars are often the source of much annoyance, because their canals, especially their posterior buccal, are sometimes difficult to find; frequently the anterior buccal canals are also difficult of approach. It is presumed that free and easy access has been secured. In extreme cases, when the canal cannot be found (generally caused by imperfect light), it is best to arrange for a second sitting; if in the meanwhile some diffusible oil, eucalyptol, for instance, has been sealed into the cavity, it is astonishing how readily a canal is found, whose location it was impossible to determine at the previous sitting. From observing the locality in which many drill, bur, or cut in looking for the entrance to the posterior buccal root-canal, I am led to believe that either our knowledge of dental anatomy is meager, or else we are under the impression that the canals originate from the pulp-chamber very much like the legs of an inverted tripod, from the bottom of the seat, in a triangle, and hence the opening is searched for too near the posterior and buccal portion of the tooth. In making cross-sections of the molar teeth at the point of bifurcation of the canals, a triangle is never found; on the contrary, the tendency is towards a straight line between the palatine and anterior buccal roots. The canal is generally slightly back of a straight line, between these two and a little nearer to the anterior buccal than the palatine.

In filling the roots of any of the lower anterior six teeth, their tendency to bifurcate should be remembered. The use of two broaches is a good practice; one broach will often not readily pass to the apex, but a finer one sometimes follows the first broach at the opening of the tooth, passing into the bifurcated portion of the root, then entering the main channel, and passes beyond the first broach to the apex of the root. This is also often the case with the upper first bicuspids.

In filling the roots of the lower bicuspids, their extreme length should be remembered; aside from that, the canals present nothing unusual; they are accessible and easily filled.

The manner of treating the lower first molar, and the position and form of the root-canals, is perhaps less perfectly understood than that of any other tooth. It is generally described as a two-rooted tooth. Remembering that the pulp-chamber and root-canals have the general outline of the tooth, the supposition that there are but two root-canals is natural. However, we do oftener find three root-canals than two. The canal of the posterior root is the most accessible, wherever the entrance to the cavity; while ready access to the anterior canals, even in badly decayed teeth, must generally be obtained, if by no other labor, at least by the cutting away of some overhanging ledge. It will then be found that whenever the canal is bifurcated the broach will pass most readily into the anterior buccal root-canal, and often the existence of an anterior lingual canal is entirely overlooked. Using three broaches is a good way to determine the location and number of the canals.

This may not be of much consequence when the canals have one common foramen, or when the broach through one of the canals passes to that foramen; but when separate foramina exist—as I have seen them—or when the broach enters the smaller of the two canals, and does not penetrate to the apex, imperfect sealing of it is almost inevitable. In the second molars we do not find this condition, and when access has been secured the filling of the root-canals is not fraught with many unfavorable possibilities.

The roots of the third molars above and below are less often treated and filled; but when this is done, the necessary manipulation proximates nearest to that required for the second molars.

The posts of pivot teeth are entirely too large, especially those of the Logan crown; on paper these posts look well, but if the root is to be cut away sufficiently to accommodate them, there will be either nothing left of it, or at least it will be weakened to a dangerous extent.

In attaching porcelain crowns to the roots of any of the six anterior teeth, whether with or without a band, no unusual difficulties are encountered; but when the first upper bicuspids are to be pivoted, the Dutch biscuit shaped construction of the root should be remembered, and also the fact borne in mind that a post can be permitted to extend only for a short distance into the root. In crowning the lower teeth, when using posts, the flattened shape of the six anterior teeth must be remembered.

For the molars above or below, all-gold caps are better suited, and the use of posts can generally be dispensed with.—*Independent Practitioner.*

